# Effects of Users’ Familiarity With the Objects Depicted in Icons on the Cognitive Performance of Icon Identification

**DOI:** 10.1177/2041669518780807

**Published:** 2018-06-11

**Authors:** Zhangfan Shen, Chengqi Xue, Haiyan Wang

**Affiliations:** School of Mechanical Engineering, Southeast University, Nanjing, China

**Keywords:** familiarity, icon identification, semantic information, complexity, icon design

## Abstract

This study investigated the effects of users’ familiarity with the objects depicted in icons on the cognitive performance of icon identification. First, without knowing the specific semantic information of icons, 20 participants were required to search for target icons among visually similar distractors for 3-hour-long training sessions across 1 week, during which their familiarity with different icons was manipulated by differential exposure frequencies. Half of the icons were presented 10 times more often than the other half. Subsequently, participants’ abilities to recall corresponding semantic information when cued with associated target icons were tested after they had learned all the icons. The results showed that, in both the visual search task and the semantic information recall task, participants performed significantly better when the icons were more familiar. Importantly, the effects of icon complexity in the visual search task diminished as participants became familiar with the icons, and the beneficial effects of familiarity in the semantic information recall task were larger when the icons were complex. These findings have practical implications for icon design. When creating new icons for time critical user interfaces, icons should be kept as simple as possible and employ familiar, commonly used, graphics.

## Introduction

With the improvement of informationization as well as the development of human–computer interactions, icons have become an important component of digital user interfaces (Li, Chen, Sha, & Lu, 2017; Nakamura & Zeng-Treitler, 2012). Compared with words, graphic symbols are able to transcend language barriers (Bocker, 1996; Caplin, 2001; Stevens, Brennan, Petocz, & Howell, 2009) and convey large amounts of information in a more concise and efficient way (Amer & Maris, 2007; Chi & Dewi, 2014; Ells & Dewar, 1979; Huang, Bias, & Schnyer, 2015; Muter & Mayson, 2007; Perry, Stevens, Wiggins, & Howell, 2008). However, people are still likely to misinterpret the meanings of icons that are poorly designed. Thus, reducing cognitive friction and improving the user experience are critical problems in icon design.

In general, when designers create an icon, three icon characteristics need to be taken into consideration: visual complexity, concreteness, and semantic distance (Garcia, Badre, & Stasko, 1994; McDougall, Curry, & de Bruijin, 1999; Silvennoinen, Kujala, & Jokinen, 2017). To date, numerous studies investigated the effects of these icon characteristics on visual search performance: people responded more quickly and more accurately to simple icons than complex icons (McDougall, de Bruijin, & Curry, 2000), users were more efficient at understanding concrete icons compared with abstract icons (Rogers & Oborne, 1987; Stammers & Hoffman, 1991), and icons with close semantic distance were easier to identify (Goonetilleke, Shih, On, & Fritsch, 2001; McDougall, Curry, & de Bruijin, 2001). However, a few researchers noticed that performance differences between different icon types diminished after users had gained considerable experience with the icons (Green & Barnard, 1990; Stotts, 1998). McDougall et al. (2000) conducted a series of experiments to examine the factors considered central to icon usability. The findings indicated that performance differences between concrete and abstract icons diminished after icon sets were used more frequently. To further explore this issue, Isherwood, McDougall, and Curry (2007) experimented with an icon identification task over a long series of trials to mimic the effects of increasing user experience. Participants were required to select the target icon (from a grid of eight icons) that they thought matched the label of semantic information. Meanwhile, they received different feedback when they chose the correct or incorrect icons. The results indicated that the importance of icon characteristics changed with user experience. Previous studies have shown familiarity to be a very important factor that has lasting effects on icon identification (e.g., Isherwood et al., 2007; McDougall et al., 2000, 2001).

**Figure 1.**
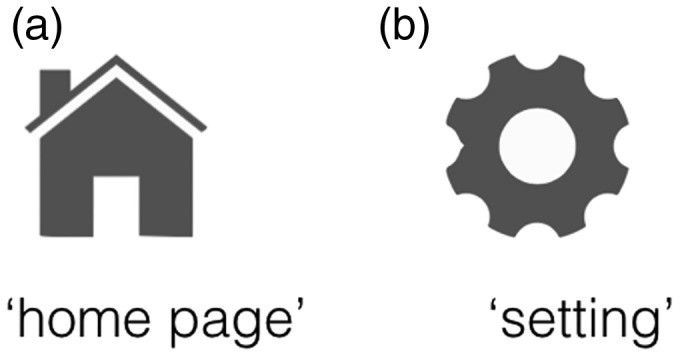
Examples of high familiarity icons.

However, there are two forms of users’ familiarity with icons (Isherwood et al., 2007). The first is familiarity with the relationship between an icon and its associated semantic information, which relates directly to its frequency of use. The second form is familiarity with the object depicted in an icon, which refers to users’ familiarity with the icon outside of any semantic context and independent of its purpose. That is to say, although a person may be familiar with the object depicted in an icon, he or she may not know the exact meaning of that icon. For example, people who have never surfed the Internet or used smart phone will be unlikely to recognize that the icons in Figure 1 refer to *home page* and *setting*. However, we can still consider these two icons as high familiarity icons because the objects depicted in them, a house and a gear, often appear in our daily life.

Unfortunately, previous studies seem to overlook the effects of the second form of familiarity. Depending on the user’s experiences in daily life, the familiarity with the objects depicted in icons might also have effects on icon identification. For example, when we see a button that we have never used before, we might understand it better and recall its function more accurately if it includes a familiar symbol. Therefore, the present study aims to investigate the role of users’ familiarity with the objects depicted in icons during icon identification.

Although only a few studies have explored the effects of familiarity on icon identification, numerous researchers have conducted extensive studies in other related areas, such as picture naming (e.g., Sirois, Kremin, & Cohen, 2006), Language learning (Groot & Keijzer, 2000), and word recognition (Coane, Balota, Dolan, & Jacoby, 2011; Meier, Reymermet, & Graf, 2013; Pazzaglia, Staub, & Rotello, 2014). Nimmo and Roodenrys (2002) found that the accuracy of immediate serial recall was better for non-word syllables that occur more frequently. Poppenk, Köhler, and Moscovitch (2010) revealed that regardless of whether familiarity was experimentally manipulated or formed by prior experience, it was easier for participants to recall familiar proverbs compared to novel ones. Almeida, Knobel, Finkbeiner, and Caramazza (2007) employed a delayed picture-naming task to test their hypothesis that stimuli frequency affected the processing of input stages. Nelson and Shffrin (2013) used a novel training paradigm to familiarize participants with new knowledge about Chinese characters and then tested participants’ performance on memory tasks. These studies demonstrated that participants' familiarity with stimuli could significantly affect their information encoding or retrieval performance. Therefore, because icon identification is a cognitive process that includes the encoding and retrieval of the associations between icons and representations, we assume that users’ familiarity with the objects depicted in icons would affect their identification performance.

To explore our prediction, in the current study, we experimentally manipulated the exposure frequency of the icons that were unknown to our participants by asking them to perform a series of visual search tasks for similar icons. As a result, participants should be more familiar with the high-frequency icons. Then, we employed a semantic information recall task to investigate the effects of familiarity with the objects depicted in icons after participants had learned all the icons. We expect that people identify familiar icons and recall corresponding semantic information more successfully. Moreover, in addition to familiarity, two more independent variables (icon complexity and concreteness) were also employed in this study; we will explore whether there are interactions between them as well.

## Method

### Ethics Statement

The procedure in this study was approved by the International Review Board of Southeast University. Participants read and signed a consent form before participating in the experiment.

### Participants

Twenty college students, 12 men and 8 women (ages ranging from 22 to 29 years) from Southeast University participated in this study. All participants were volunteers and had never participated in similar experiments before.

### Materials and Design

Three hundred and eighty icons were chosen from the symbols used on industrial machineries and household goods as well as from the icons used in the systems of computers and smart phones. Before the formal experiments, we recruited 50 volunteers to rate all the three factors (complexity, concreteness, and familiarity) of each icon on a 5-point scale. Instructions were similar to those adopted in previous studies (Gilhooly & Logie, 1980; McDougall et al., 1999, 2000; Snodgrass & Vanderwart, 1980). For complexity ratings, icons were to be regarded as complex if they contained a large amount of detail or intricacy (1 = *definitely simple*, 5 = *definitely complex*); for concreteness ratings, icons were to be regarded as concrete if they depicted real objects (1 = *definitely abstract*, 5 = *definitely concrete*); for familiarity ratings, icons were to be regarded as familiar if they often appeared in daily life (1 = *definitely unfamiliar*, 5 = *definitely familiar*). Based on the rating results, 80 icons from four sets were used in the experiment (20 complex and abstract icons, 20 complex and concrete icons, 20 simple and abstract icons, and 20 simple and concrete icons, see Appendix). Subsequently, one-way analyses of variance (ANOVAs), followed by Newman-Keuls comparisons, were conducted to ensure the ratings differed in accordance with the requirements of each experimental condition (McDougall et al., 2000). The results indicated that there were significant differences between the complexity ratings of the complex group and the simple group, *F*(1, 78) = 734.67, *p* < .001, and between the concreteness ratings of the concrete group and the abstract group, *F*(1, 78) = 1,553.59, *p* < .001. However, there was no significant difference among the familiarity ratings of icons, *F*(79, 3920) = 1.19, *p* = .19, or icons groups, *F*(3, 76) = .74, *p* = .44. Table 1 shows the mean ratings and standard deviations for complexity, concreteness, and familiarity in each icon set.

**Table 1.** Mean Ratings and Standard Deviations for Complexity, Concreteness, and Familiarity in Each Icon Set.

Icon set	Icon characteristic					
	Complexity		Concreteness		Familiarity	
	Mean	*SD*	Mean	*SD*	Mean	*SD*
Complex & Abstract	3.92	0.29	1.67	0.26	2.98	0.73
Complex & Concrete	4.22	0.38	4.31	0.32	3.17	0.70
Simple & Abstract	1.80	0.42	1.33	0.22	3.04	0.65
Simple & Concrete	1.87	0.27	4.38	0.35	3.11	0.61

Subsequently, each icon was assigned a Chinese word as a piece of semantic information which offered a relevant description of the paired icon. The assigning of semantic information to icons was based on the agreement between five experts (icon designers). To ensure that the icon/semantic information pairs used in our experiment were at the same level, we asked 20 volunteers to rate the semantic distance of each icon/semantic information pair (1 = *not closely related*, 5 = *very strongly related*). Semantic distance is a measure of the closeness of the relationship between the icon and what it is intended to represent (McDougall et al., 1999). The mean value of the ratings was 3.22, and the standard deviation value was 0.52. The result of ANOVA showed that there was no significant difference in semantic distance among icon/semantic information pairs, *F*(79, 1520) = 1.68, *p* = .25.

**Figure 2.**
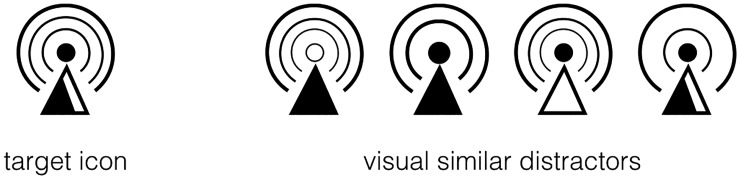
Examples of target icon and visual similar distractors.

Finally, without changing the overall shape of each icon, we modified some details (e.g., adding or deleting lines, enlarging or narrowing internal elements, changing filling areas, etc.) and created four distractors for each icon (e.g., see Figure 2). By using these distractors, participants had to encode the entire icon rather than a subset of features in the visual search task.

### Visual Search Task

**Figure 3.**
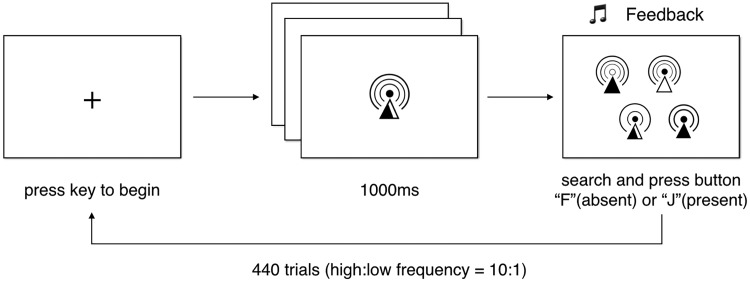
Trial sequence for visual search task.

Participants performed a visual search task during three sessions on three different days in a week, and each session consisted of 440 trials (see Figure 3). A 5-minute break was given during one experimental session. For each participant, there were 80 icons (20 complex & abstract icons, 20 complex & concrete icons, 20 simple & abstract icons, and 20 simple & concrete icons) in total. Taking the icon set of complex & abstract as an example, 20 icons were randomly assigned into high- and low-frequency groups such that 10 icons were high-frequency icons and the other 10 were low-frequency icons. As the ratio of high versus low frequency was fixed to be 10:1, and therefore, by using these 20 icons, we had 100 high-frequency trials (that is, 10 × 10 = 100) and 10 low frequency trials (that is, 10 × 1 = 10). Same procedure was applied to the rest three groups. Altogether, we had 400 high-frequency trials (100 complex & abstract trials, 100 complex & concrete trials, 100 simple & abstract trials, and 100 simple & concrete trials) and 40 low-frequency trials (10 complex & abstract trials, 10 complex & concrete trials, 10 simple & abstract trials, and 10 simple & concrete trials), making total of 440 trials. Each experimental trial began with a fixation, and participants had to press any button to continue. Participants saw a randomly selected target icon in the center of the screen for 1 second, which was followed by a display of four similar icons. Participants were required to respond as to whether the target icon was present or absent. After participants pressed a button, they received auditory feedback that indicated whether their response was correct or not. The target icon was present in the search array in half of the trials. The trial order and whether the target icon was present or not in each trial were randomly determined for each subject and session. The dependent variables were accuracy and response times in reporting whether the target was present or absent in each trial.

### Semantic Information Recall Task

**Figure 4.**
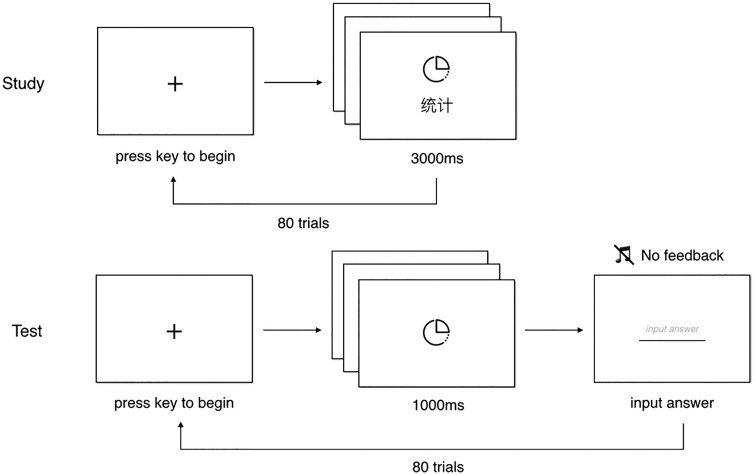
Trial sequence for semantic information study and test.

After we familiarized participants with 80 icons over a 3-hour-long training (the visual search task with three sessions), with half of the icons exposed more frequently, each participant learned the relationships between icons and semantic information one by one in random order. Subsequently, participants’ abilities to recall the semantic information were tested (see Figure 4). In the study session, each experimental trial began with a fixation, and participants had to press any button to continue. Participants saw a randomly selected target icon and associated semantic information for 3 seconds before the next trial appeared. The icon-semantic information pairs were same for all participants. In the test session, a cued-recall test was given after participants had learned all the icons and sematic information. A randomly selected icon appeared in the center of the screen for 1 second, followed by a display of the input box. Participants had to recall the semantic information associated with the icon and enter it in the input box. There was no time limit, and participants did not receive any auditory feedback during this task. We instructed participants to try their best to be as accurate as possible in recalling and entering the semantic information, and we only measured accuracy in this task.

## Results

We analyzed response time (RT) by linear mixed-effects regression, and we analyzed accuracy data via logistic mixed-effects regressions (Baayen, Davidson, & Bates, 2008; Jaeger, 2008). For the RT analyses of the visual search task, we considered only correct trials (9.3% error). Then, we excluded from the analyses cases with RTs greater than 3 median absolute deviations above or below the median RT, calculated separately for each participant, session and condition (3.4%).

### Visual Search Task

**Figure 5.**
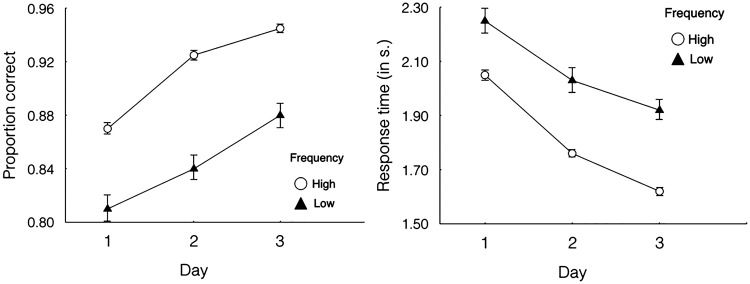
Mean performance on visual search task trials for low- and high-frequency icons over 3 days of training. Left panel shows accuracy and right panel shows response times. Error bars indicate ± 1 *SE*s.

Over the 3 days of training, participants performed better on the visual search task (see Figure 5), becoming faster, ΔAIC [akaike information criterion] = −1,820, LLR [log likelihood ratio] χ^2^(1) = 1,822.312, *p* < .001, and more accurate, ΔAIC = −270, LLR χ^2^(1) = 272.719, *p* < .001, in identifying whether the target icon was present or absent from the search display. Importantly, participants identified high-frequency icons more quickly, ΔAIC = −229, LLR χ^2^(1) = 230.798, *p* < .001, and more accurately, ΔAIC = −118, LLR χ^2^(1) = 119.593, *p* < .001. Participants’ accuracy and response times on the visual search task improved significantly for both frequency conditions as all the icons became more familiar. In addition, performance was always better for the high-frequency icons.

**Figure 6.**
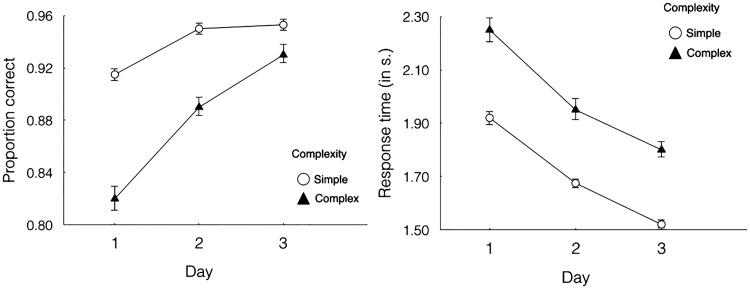
Mean performance on visual search task trials for simple and complex icons over 3 days of training. Left panel shows accuracy and right panel shows response times. Error bars indicate ± 1 *SE*s.

Figure 6 shows the mean performance on visual search task trials for simple and complex icons over 3 days of training. Participants became faster, ΔAIC = −1,090, LLR χ^2^(1) =1,092.390, *p* < .001, and more accurate, ΔAIC = −194, LLR χ^2^(1) = 195.959, *p* < .001, when they were searching for simple target icons. In addition, a strong interaction was observed between the effects of icon complexity and blocks of trials on search accuracy, ΔAIC = −11, LLR χ^2^(1) = 13.032, *p* < .001. However, no interaction was found between complexity and blocks of trials on response times, ΔAIC = 2, LLR χ^2^(1) = 0.068, *p* = .795.

**Figure 7.**
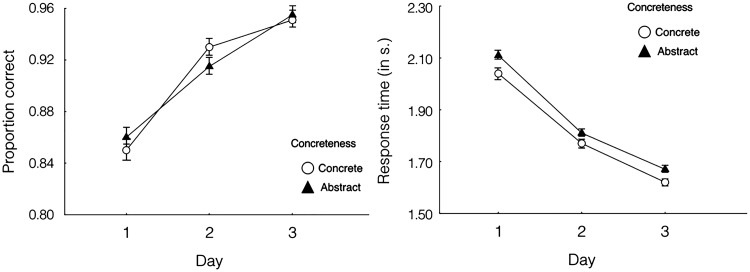
Mean performance on visual search task trials for concrete and abstract icons over 3 days of training. Left panel shows accuracy and right panel shows response times. Error bars indicate ± 1 *SE*s.

Figure 7 shows the mean performance on visual search task trials for concrete and abstract icons over 3 days of training. There was no effect of icon concreteness on search accuracy, ΔAIC = 2, LLR χ^2^(1) = 0.152, *p* = .697. However, participants responded more quickly to concrete icons, ΔAIC = −42, LLR χ^2^(1) = 43.932, *p* < .001. In addition, there was no significant interaction between icon concreteness and blocks of trials on accuracy, ΔAIC = 2, LLR χ^2^(1) = 0.118, *p* = .731, or response times, ΔAIC = 2, LLR χ^2^(1) = 0.008, *p* = .931.

### Semantic Information Recall Task

**Figure 8.**
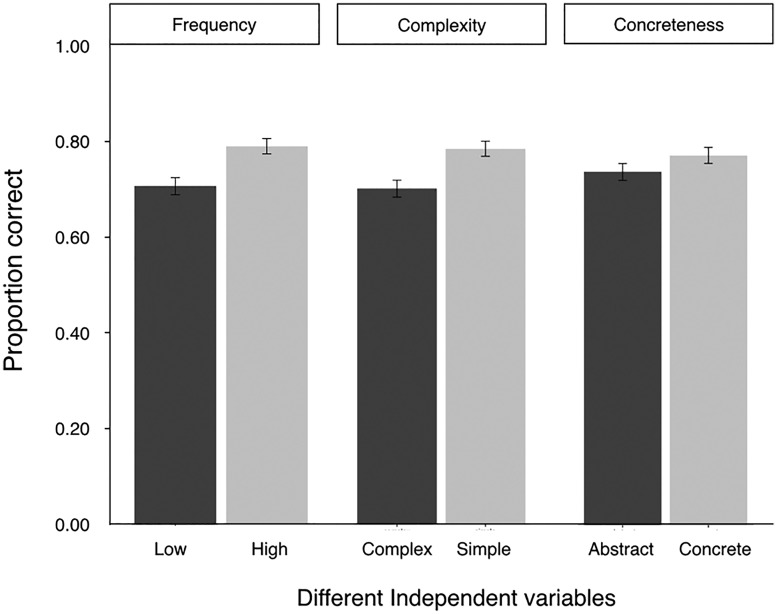
Mean performance on semantics recall task trials in different conditions. Error bars indicate ± 1 *SE*s.

Figure 8 shows the mean performance on semantic information recall task trials in different conditions. Participants performed significantly better when the icons were more familiar, ΔAIC = −10.4, LLR χ^2^(1) = 11.651, *p* < .001, and when the icons were simple, ΔAIC =−10.7, LLR χ^2^(1) = 12.690, *p* < .001. However, there was no significant effect of icon concreteness on recall accuracy, ΔAIC = −1, LLR χ^2^(1) = 2.099, *p* = .147.

**Figure 9.**
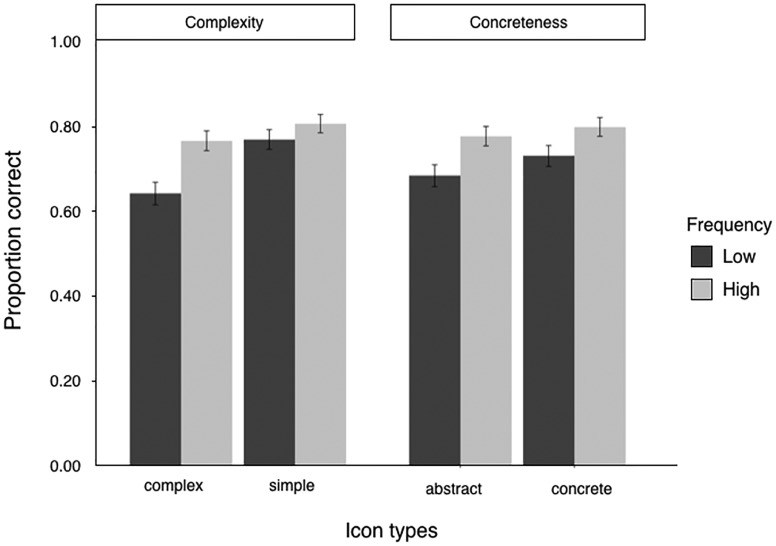
Mean performance on semantics recall task trials for low and high-frequency icons in two different conditions (different complexity conditions and different concreteness conditions). Error bars indicate ± 1 *SE*s.

Figure 9 shows the mean performance on semantic information recall task trials for low- and high-frequency icons in different conditions (different complexity conditions and different concreteness conditions). There were significant effects of familiarity for complex icons, ΔAIC = −11, LLR χ^2^(1) = 12.768, *p* < .001; abstract icons, ΔAIC = −5, LLR χ^2^(1) =7.361, *p* < .01; and concrete icons, ΔAIC = −2, LLR χ^2^(1) = 4.375, *p* < .05. However, there was no significant effect of familiarity for simple icons, ΔAIC = 1, LLR χ^2^(1) = 1.397, *p* = .237. In addition, a strong interaction was observed between the effects of icon complexity and familiarity, ΔAIC = −10, LLR χ^2^(1) = 12.690, *p* < .001. However, there was no significant interaction between icon concreteness and familiarity, ΔAIC = 2, LLR χ^2^(1) = 2.239, *p* = .326.

**Figure 10.**
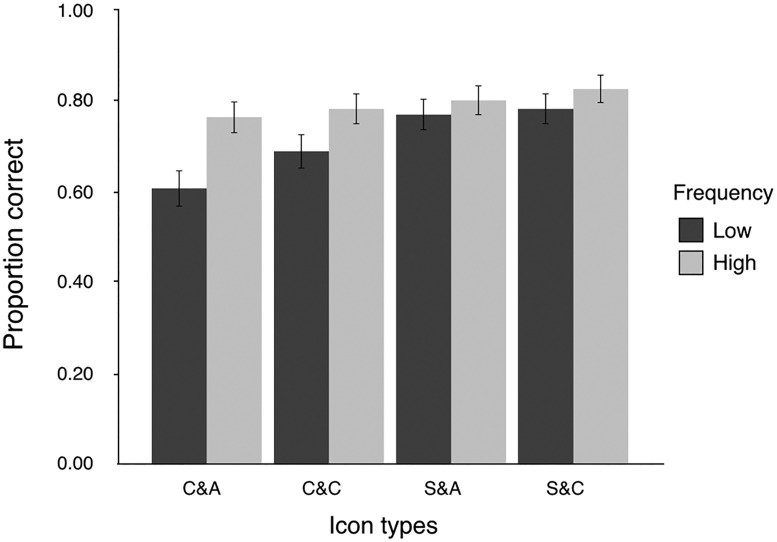
Mean performance on semantics recall task trials for high- and low-frequency icons of different icon types. Error bars indicate ± 1 *SE*s. C&A = complex and abstract icons; C&C = complex and concrete icons; S&A = simple and abstract icons; S&C = simple and concrete icons.

Figure 10 shows the mean performance on semantic information recall task trials for low- and high-frequency icons for four different icon types. There were significant effects of familiarity for complex and abstract icons, ΔAIC = −10, LLR χ^2^(1) = 10.485, *p* < .001, and for complex and concrete icons, ΔAIC = −4, LLR χ^2^(1) = 3.366, *p* < .05. However, there was no significant effect of familiarity for simple and abstract icons, ΔAIC = 2, LLR χ^2^(1) = 0.462, *p* = .497, or for simple and concrete icons, ΔAIC = 1, LLR χ^2^(1) = 0.981, *p* = .322.

## Discussion

### Visual Search Task

The key question of this study is how to experimentally simulate users’ different levels of familiarity with the objects depicted in icons. According to the familiarity encoding methods used in related areas (Almeida et al., 2007; Nelson & Shffrin, 2013), we manipulated participants’ familiarity with icons and created familiarity differences between high-frequency icons and low-frequency icons by controlling the exposure frequency of the icons in a visual search task. Compared with the visual search tasks used in previous studies, the current study employed a stricter experimental design: (a) Instead of using different target icons as distractors, we created visually similar icons to increase the difficulty of the task. Thus, in order to find the target, participants had to encode the entire icon, which avoided participants’ exorbitant accuracy and too-quick responses based on the difference of any obvious feature. (b) To minimize the pre-exiting familiarity differences between icons, high- and low-frequency icons were randomly selected for each participant. (c) Half of the icons were exposed 10 times more often than the other half. As a result, the familiarity difference between high-frequency and low-frequency icons should be obvious after three days of training.

Most results of the visual search task were consistent with the results of previous studies (Isherwood et al., 2007; McDougall et al., 2000, 2001), suggesting that the visual complexity of icons had significant effects on response times and that visual search performance improved with user experience. However, in the current study, because icon familiarity was manipulated by different exposure frequencies, we observed the effects of icon familiarity more clearly (see Figure 3). Participants’ visual search performance was definitely faster and more accurate when the icons were from high-frequency groups. Here, we provide more direct evidence demonstrating that icon familiarity significantly affects visual search performance.

Moreover, McDougall et al. (2000) only analyzed the data of response times because the participants in their experiment made a minimal number of errors. Therefore, the effect of icon familiarity on visual search accuracy was still unknown. To resolve this issue, as mentioned earlier, we appropriately increased the difficulty of the visual search task. As predicted, we obtained the data on proportion correctly, and the results indicated that visual complexity also had a significant effect on visual search accuracy (see Figure 4).

In addition, our results showed that there was a significant interaction on the accuracy of the visual search task. The differences in accuracy between simple and complex icons diminished greatly as participants became familiar with the icons. The reason for this, we contend, is that as the exposure times of the complex icons increased, participants became more familiar with the features of each complex icon. As a result, it became less difficult for them to identify the target icons, and the error rate declined significantly. Besides, since the simple icons contained fewer features, search performance for simple icons was still better than that for complex icons and tended to stabilize with time. However, there was no interaction effect in response time of the visual search task. The differences in response time between simple and complex icons were relatively constant throughout the training session. It is most likely due to that the training session was not enough. The left panel of Figure 6 shows that the increasing of accuracy after the Day 2 was very little, which appeared a ceiling effect. However, the response time of simple icons kept decreasing after the second session, implying the inadequacy of training (see the right panel of Figure 6). We instructed participants to identify target icons as accurate as possible, and there was no time limit in the experiment. Thus, the training needed to improve RT would be longer than that for accuracy. Therefore, we would expect to see the interaction effect in response time as well if the participants were provided sufficient training.

Finally, our results also showed that although there was no effect of icon concreteness on visual search accuracy, people responded more quickly to concrete icons than to abstract icons (see Figure 5). One possible explanation is that concrete icons contain more objects that people often see in their daily lives, and it might be easier for people to identify icons that include more familiar elements.

### Semantic Information Recall Task

After numerous trials of familiarity training with icons that differed in terms of their familiarity, the effects of icon familiarity in the semantic information recall task were expected to be observed more directly. Our results revealed that users’ familiarity with the objects depicted in icons indeed influenced icon identification. As predicted, participants performed significantly better when the icons were more familiar. According to the theory of Johnson, Paivio, and Clark (1996), the processing of semantic information recall can be divided into three steps: (a) search for and locate the icon, then preliminarily identify the object depicted; (b) retrieve relevant information stored in one’s memory based on the results of identification; and (c) activate the correct semantic information associated with the icon. We contend that users’ familiarity with the objects depicted in icons may influence one or more steps in the cognitive processing described earlier.

Numerous researchers have suggested that the currently active information in an individual’s working memory will decay gradually with time, and it will become unavailable to subsequent processing if it is not reactivated (Baddeley, Thomson, & Buchanan, 1975; Camos, Lagner, & Barrouillet, 2009; Schweickert & Boruff, 1986). From this point of view, Working Memory is limited by how often activation can be rehearsed and by how quickly they decay. If reactivation can occur more frequently, it might prevent item activation from decaying or reduce decay rate greatly. In the semantic information task, all the relative representations were held in memory and began decaying after participants finished encoding the relationships between icons and the semantic information. We contend one possible explanation is that compared to more familiar icons, less familiar icons were relatively more difficult to reactivate, then the related semantic information decayed faster, hurting recall performance as a result. Moreover, spreading activation might be a more likely explanation of the frequency effects on semantic recall. The theory of spreading activation assumes that retrieval of information from declarative memory is governed by information activations, and items with higher activations can be retrieved more accurately and faster than items with lower activations (Anderson, 1983; Collins & Loftus, 1988). Besides, activation could also spread over a semantic network rapidly, from one item to associative relevancy (Anderson & Pirolli, 1984; Anderson, Reder & Lebiere, 1996). According to this theory, the high-frequency icons spread more activation than the low-frequency icons in the visual search task. Correspondingly, the icons with higher activation spread more activation to the associated semantic information, which resulted in better performance in the semantic recall task. On the contrary, other researchers believe that the representations do not decay on their own but that attempting to hold a large amount of information actively in one’s working memory results in interference, such as confusion, competition, or feature overlap, which hinders the retrieval of information (Nairne, 1990; Oberauer & Kliegl, 2006; Oberauer, Lewandowsky, Farrell, Jarrold, & Greaves et al., 2012; Saito & Miyake, 2004). Within this framework, we contend that familiarity with the icons played an important role at the learning stage. If the icons were more familiar, the relationships between the icons and the semantic information were better encoded, and thus the representations were stronger and less susceptible to interference. Meanwhile, if the icons were less familiar, especially those containing more complex visual details, the relationships between those icons and their semantic information became relatively weak, which likely accounts for the participants’ impaired recall performance.

In addition, the results also showed that icon complexity was another important factor that influenced participants’ semantic information recall performance. The explanation might be similar to that concerning the effects of familiarity, as discussed earlier: The information of complex icons was more difficult to reactivate and decayed faster as a result, or the relationships between complex icons and semantic information were not encoded successfully during the learning stage, and thus, they were more susceptible to interference. However, it should be noted that we did not observe a significant effect of concreteness, which Isherwood et al. (2007) identified in their study. The reason for this, we believe, is the difference between experimental paradigms. In the experiment of Isherwood et al. (2007), participants were required to select the icons that they thought matched the semantic information, but without learning the correct meaning of the icons. In contrast, in our study, participants completed a study session before they took the semantic information recall test.

Moreover, our results also showed that there was a significant interaction between complexity and familiarity (see Figure 7). When the icons were simple, the recall accuracy for both high-frequency icons and low-frequency icons was high. However, when the icons became complex, the participants’ accuracy with low-frequency icons decreased greatly, while their accuracy with high-frequency icons could still be maintained at a high level. That is to say, the beneficial effects of familiarity in the semantic information recall task increased when the icons became complex. Thus, in a sense, improving icon familiarity can reduce the performance difference caused by icon complexity. A similar phenomenon can also be observed in Figure 8. The reason is that compared to the other three types of icons, complex and abstract icons were most difficult for participants to identify and recall.

### Implications for Design

These findings have important implications for the design of icons. (a) If visual search activity is the key task of the digital interface, and the operator’s response time seriously affects the quality of the entire mission, for example, in aircraft operations, nuclear power control, and weapons operation, then reducing icon complexity should be a top priority. The reason is that the effect of icon complexity on reaction time does not diminish as users receive extra training. Thus, in this case, designers should reduce the amount of details in icons to lessen the visual complexity. (b) If the task is not time-critical and the context in which the digital interface used is relatively relaxed, the designer may increase the level of details in icons to make the whole interface more attractive to users. Although the improvement of icon complexity might influence the identification of the icons at the beginning, that effect will quickly diminish as a result of user experience. (c) Designers should not merely consider the user's familiarity with the relationship between an icon and its meaning; the effect of the icon’s familiarity should also be taken into consideration when designers create new icons. Icons containing more familiar symbols are easier to remember and identify. Thus, unless the design of a new icon is very simple, in most cases, it is better for designers to use common graphics to create new icons.

## Conclusions

Users’ familiarity with icons takes two forms: familiarity with the relationships between icons and semantic information, and familiarity with the objects depicted in icons. Although numerous studies have demonstrated that the former is one of the primary determinants of icon identification, it is still not known whether the latter affects the cognitive process. In this article, we investigated the effects of users’ familiarity with the objects depicted in icons on the cognitive performance of icon identification. The results showed that, in both visual search task and the semantic information recall task, participants performed worse when the objects depicted in icons were less familiar. Importantly, the detrimental effects of low familiarity on recall performance increased as the complexity of icon increased. These findings extend our understanding of how familiarity affects icon identification. However, this study is limited in several aspects. (a) Due to the limitation of experimental equipment, we only analyzed the accuracy data of the semantic information recall task. More data such as eye movement data and electroencephalograms (EEGs) can be collected to provide more convincing evidence. (b) Participants in this study are all college students who have rich experience of using human-computer interfaces. However, the user groups should be diverse in reality, including children, elder, etc. In the future, we will employ more advanced experimental equipment and expand our study to more participants and more participant diversity.

## Appendix

**Icons Used in the Experiment**

## References

[bibr1-2041669518780807] Almeida J. Knobel M. Finkbeiner M. Caramazza A. (2007) The locus of the frequency effect in picture naming: When recognizing is not enough. Psychonomic Bulletin & Review 14: 1177–1182. 18229493 10.3758/bf03193109

[bibr2-2041669518780807] Amer T. S. Maris J. M. B. (2007) Signal words and signal icons in application control and information technology exception messages—Hazard matching and habituation effects. Journal of Information Systems 21: 1–26.

[bibr3-2041669518780807] Anderson J. R. (1983) A spreading activation theory of memory. Journal of Verbal Learning and Verbal Behavior 22: 261–295.

[bibr4-2041669518780807] Anderson J. R. Pirolli P. L. (1984) Spread of activation. Journal of Experimental Psychology Learning Memory & Cognition 10: 791–798.

[bibr5-2041669518780807] Anderson J. R. Reder L. M. Lebiere C. (1996) Working memory: Activation limitations on retrieval. Cognitive Psychology 30: 221–256. 8660785 10.1006/cogp.1996.0007

[bibr6-2041669518780807] Baayen R. H. Davidson D. J. Bates D. M. (2008) Mixed-effects modeling with crossed random effects for subjects and items. Journal of Memory and Language 59: 390–412.

[bibr7-2041669518780807] Baddeley A. D. Thomson N. Buchanan M. (1975) Word length and the structure of memory. Journal of Verbal Learning & Verbal Behavior 14: 575–589.

[bibr8-2041669518780807] Bocker M. (1996) A multiple index approach for the evaluation of pictograms. Computer Standards and Interfaces 18: 107–115.

[bibr9-2041669518780807] Camos V. Lagner P. Barrouillet P. (2009) Two maintenance mechanisms of verbal information in working memory. Journal of Memory and Language 61: 457–469.

[bibr10-2041669518780807] Caplin S. (2001) Icon design: Graphic icons in computer interface design, London, England: Cassell.

[bibr11-2041669518780807] Chi C. F. Dewi R. S. (2014) Matching performance of vehicle icons in graphical and textual formats. Applied Ergonomics 45: 904–916. 24315463 10.1016/j.apergo.2013.11.009

[bibr12-2041669518780807] Coane J. H. Balota D. A. Dolan P. O. Jacoby L. L. (2011) Not all sources of familiarity are created equal: The case of word frequency and repetition in episodic recognition. Memory & Cognition 39: 791–805. 21264634 10.3758/s13421-010-0069-5

[bibr13-2041669518780807] Collins A. M. Loftus E. F. (1988) A spreading-activation theory of semantic processing. Readings in Cognitive Science 82: 126–136.

[bibr14-2041669518780807] Ells J. G. Dewar R. E. (1979) Rapid comprehension of verbal and symbolic traffic sign messages. Human Factors 21: 161–168.

[bibr15-2041669518780807] Garcia M. Badre A. N. Stasko T. (1994) Development and validation of icons varying in their abstractness. Interacting With Computers 6: 191–211.

[bibr16-2041669518780807] Gilhooly K. J. Logie R. H. (1980) Age-of-acquisition, imagery, concreteness, familiarity, and ambiguity measures for 1,944 words. Behavior Research Methods & Instrumentation 12: 395–427.

[bibr17-2041669518780807] Goonetilleke R. S. Shih H. M. On H. K. Fritsch J. (2001) Effects of training and representational characteristics in icon design. International Journal of Human-Computer Studies 55: 741–760.

[bibr18-2041669518780807] Green, A. J. K., & Barnard, P. J. (1990). Iconic interfacing: The role of icon distinctiveness and fixed or variable screen locations. In D. Diaper, D. Gilmore, G. Cockton, & B. Shackel (Eds.), *Human – Computer Interaction – Interact ‘90* (pp. 457–462). Amsterdam: Elsevier Science.

[bibr19-2041669518780807] Groot A. M. B. D. Keijzer R. (2000) What is hard to learn is easy to forget: The roles of word concreteness, cognate status, and word frequency in foreign-language vocabulary learning and forgetting. Language Learning 50: 1–56.

[bibr20-2041669518780807] Huang S. C. Bias R. G. Schnyer D. (2015) How are icons processed by the brain? Neuroimaging measures of four types of visual stimuli used in information systems. Journal of the Association for Information Science & Technology 66: 702–720.

[bibr21-2041669518780807] Isherwood S. J. Mcdougall S. J. P. Curry M. B. (2007) Icon identification in context: The changing role of icon characteristics with user experience. Human Factors 49: 465–476. 17552310 10.1518/001872007X200102

[bibr22-2041669518780807] Jaeger T. F. (2008) Categorical data analysis: Away from ANOVAs (transformation or not) and towards logit mixed models. Journal of Memory and Language 59: 434–446. 19884961 10.1016/j.jml.2007.11.007 PMC2613284

[bibr23-2041669518780807] Johnson C. J. Paivio A. Clark J. M. (1996) Cognitive components of picture naming. Psychological Bulletin 120: 113–139. 8711012 10.1037/0033-2909.120.1.113

[bibr24-2041669518780807] Li R. Chen Y. V. Sha C. Lu Z. (2017) Effects of interface layout on the usability of in-vehicle information systems and driving safety. Displays 49: 123–132.

[bibr25-2041669518780807] McDougall S. J. P. Curry M. B. de Bruijin O. (1999) Measuring symbol and icon characteristics: Norms for concreteness, complexity, meaningfulness, familiarity, and semantic distance for 239 symbols. Behavior Research Methods Instruments & Computers 31: 487–519. 10.3758/bf03200730 10502873

[bibr26-2041669518780807] McDougall S. J. P. Curry M. B. de Bruijin O. (2001) The effects of visual information on users’ mental models: An evaluation of pathfinder analysis as a measure of icon usability. International Journal of Cognitive Ergonomics 5: 59–84.

[bibr27-2041669518780807] McDougall S. J. P. de Bruijin O. Curry M. B. (2000) Exploring the effects of icon characteristics on user performance: The role of icon concreteness, complexity, and distinctiveness. Journal of Experimental Psychology Applied 6: 291–306. 11218339 10.1037//1076-898x.6.4.291

[bibr28-2041669518780807] Meier B. Reymermet A. Rothen N. Graf P. (2013) Recognition memory across the lifespan: The impact of word frequency and study-test interval on estimates of familiarity and recollection. Frontiers in Psychology 4: 787. 24198796 10.3389/fpsyg.2013.00787 PMC3812907

[bibr29-2041669518780807] Muter P. Mayson C. (1986) The role of graphics in item selection from menus. Behaviour & Information Technology 5: 89–95.

[bibr30-2041669518780807] Nakamura C. Zeng-Treitler Q. (2012) A taxonomy of representation strategies in iconic communication. International Journal of Human-Computer Studies 70: 535–551. 22754274 10.1016/j.ijhcs.2012.02.009 PMC3383821

[bibr31-2041669518780807] Nairne J. S. (1990) A feature model of immediate memory. Memory & Cognition 18: 251–269. 2192233 10.3758/bf03213879

[bibr32-2041669518780807] Nelson A. Shiffrin R. (2013) The co-evolution of knowledge and event memory. Psychological Review 120: 356–394. 23458086 10.1037/a0032020

[bibr33-2041669518780807] Nimmo L. M. Roodenrys S. (2002) Syllable frequency effects on phonological short-term memory tasks. Applied Psycholinguistics 23: 643–660.

[bibr34-2041669518780807] Oberauer K. Kliegl R. (2006) A formal model of capacity limits in working memory. Journal of Memory & Language 55: 601–626.

[bibr35-2041669518780807] Oberauer K. Lewandowsky S. Farrell S. Jarrold C. Greaves M. (2012) Modeling working memory: An interference model of complex span. Psychonomic Bulletin & Review 19: 779–819. 22715024 10.3758/s13423-012-0272-4

[bibr36-2041669518780807] Pazzaglia A. M. Staub A. Rotello C. M. (2014) Encoding time and the mirror effect in recognition memory: Evidence from eye-tracking. Journal of Memory & Language 75: 77–92.

[bibr37-2041669518780807] Perry N. C. Stevens C. J. Wiggins M. W. Howell C. E. (2008) Cough once for danger: Icons versus abstract warnings as informative alerts in civil aviation. Human Factors the Journal of the Human Factors & Ergonomics Society 49: 1061–1071. 10.1518/001872007X249929 18074705

[bibr38-2041669518780807] Poppenk J. Köhler S. Moscovitch M. (2010) Revisiting the novelty effect: When familiarity, not novelty, enhances memory. Journal of Experimental Psychology: Learning, Memory, and Cognition 36(5): 1321–1330. doi: 10.1037/a0019900. 10.1037/a0019900 20804299

[bibr39-2041669518780807] Rogers Y. Oborne D. J. (1987) Pictorial communication of abstract verbs in relation to human-computer interaction. British Journal of Psychology 78: 99–112.

[bibr40-2041669518780807] Saito S. Miyake A. (2004) On the nature of forgetting and the processing–storage relationship in reading span performance. Journal of Memory & Language 50: 425–443.

[bibr41-2041669518780807] Schweickert R. Boruff B. (1986) Short-term memory capacity: Magic number or magic spell? Journal of Experimental Psychology Learning Memory & Cognition 12: 419–425. 10.1037//0278-7393.12.3.419 2942626

[bibr42-2041669518780807] Silvennoinen J. M. Kujala T. Jokinen J. (2017) Semantic distance as a critical factor in icon design for in-car infotainment systems. Applied Ergonomics 65: 369–381. 28802458 10.1016/j.apergo.2017.07.014

[bibr43-2041669518780807] Sirois M. Kremin H. Cohen H. (2006) Picture-naming norms for Canadian French: Name agreement, familiarity, visual complexity, and age of acquisition. Behavior Research Methods 38: 300–306. 16956106 10.3758/bf03192781

[bibr44-2041669518780807] Snodgrass J. G. Vanderwart M. (1980) A standardized set of 260 pictures: Norms for name agreement, image agreement, familiarity, and visual complexity. Journal of Experimental Psychology Human Learning & Memory 6: 174–215. 7373248 10.1037//0278-7393.6.2.174

[bibr45-2041669518780807] Stammers R. Hoffman J. (1991) Transfer between icon sets and ratings of icon concreteness and appropriateness. Human Factors & Ergonomics Society Annual Meeting Proceedings 35: 354–358.

[bibr46-2041669518780807] Stevens C. J. Brennan D. Petocz A. Howell C. (2009) Designing informative warning signals: Effects of indicator type, modality, and task demand on recognition speed and accuracy. Advances in Cognitive Psychology 5: 84–90. 20523852 10.2478/v10053-008-0064-6 PMC2865004

[bibr47-2041669518780807] Stotts D. B. (1998) The usefulness of icons on the computer interface: Effect of graphical abstraction and functional representation on experienced and novice users. Proceedings of the Human Factors & Ergonomics Society Annual Meeting 42: 453–457.

