# CORRIGENDUM to “Effects of Users’ Familiarity With the Objects Depicted in Icons on the Cognitive Performance of Icon Identification”

**DOI:** 10.1177/2041669520933026

**Published:** 2020-05-28

Shen, Z., Xue, C., & Wang, H. (2018). Effects of Users’ Familiarity With the Objects Depicted in Icons on the Cognitive Performance of Icon Identification. *i-Perception*, *9*(3), 1–17. https://doi.org/10.1177/2041669518780807

The authors regret that the following admission was erroneously not included in the article. The experimental paradigm used in this study was developed in Professor Lynne Reder’s lab, as previously published in Shen, Popov, Delahay & Reder (2018). The study’s methods and design were based on those ideas. The following reference should have been included for transparency:

Shen, Z., Popov, V., Delahay, A. B., & Reder, L. M. (2018). Item strength affects working memory capacity. *Memory & Cognition*, *46*(2), 204–215. https://doi.org/10.3758/s13421-017-0758-4

The authors apologise for this omission.

